# DAPPER: a data-mining resource for protein-protein interactions

**DOI:** 10.1186/s13040-015-0063-3

**Published:** 2015-09-24

**Authors:** Syed Haider, Zoltan Lipinszki, Marcin R. Przewloka, Yaseen Ladak, Pier Paolo D’Avino, Yuu Kimata, Pietro Lio’, David M. Glover

**Affiliations:** 1 Computer Laboratory, University of Cambridge, Cambridge, CB3 0FD UK; 2 Department of Genetics, University of Cambridge, Downing Street, Cambridge, CB2 3EH UK; 3 Oxford Centre for Integrative Systems Biology, Department of Biochemistry, University of Oxford, Oxford, OX1 3QU UK; 4 Department of Pathology, University of Cambridge, Tennis Court Road, Cambridge, CB2 1QP UK

**Keywords:** Proteomics data mining, Protein-protein interactions, Protein complexes, Mass spectrometry, Data-integration, *Drosophila melanogaster*

## Abstract

**Background:**

The identification of interaction networks between proteins and complexes holds the promise of offering novel insights into the molecular mechanisms that regulate many biological processes. With increasing volumes of such datasets, especially in model organisms such as *Drosophila melanogaster*, there exists a pressing need for specialised tools, which can seamlessly collect, integrate and analyse these data. Here we describe a database coupled with a mining tool for protein-protein interactions (DAPPER), developed as a rich resource for studying multi-protein complexes in *Drosophila melanogaster*.

**Results:**

This proteomics database is compiled through mass spectrometric analyses of many protein complexes affinity purified from *Drosophila* tissues and cultured cells. The web access to DAPPER is provided via an accelerated version of BioMart software enabling data-mining through customised querying and output formats. The protein-protein interaction dataset is annotated with FlyBase identifiers, and further linked to the Ensembl database using BioMart’s data-federation model, thereby enabling complex multi-dataset queries. DAPPER is open source, with all its contents and source code are freely available.

**Conclusions:**

DAPPER offers an *easy-to-navigate* and extensible platform for real-time integration of diverse resources containing new and existing protein-protein interaction datasets of *Drosophila melanogaster*.

**Electronic supplementary material:**

The online version of this article (doi:10.1186/s13040-015-0063-3) contains supplementary material, which is available to authorized users.

## Background

Proteins control cellular events in living organisms by catalysing biochemical reactions, transporting molecules, providing scaffolds for distinct pathways, and receiving and transmitting signals from the environment. The identification of protein interaction networks can offers insights into the molecular mechanisms that regulate many biological processes. Proteomics allows large-scale analysis of protein-protein interactions in an efficient and sensitive way. Targeted and high throughput approaches now enable the identification of protein complexes [1, 2], entire interaction networks (reviewed in reference [3]), and post-synthetic modifications that regulate protein-protein interactions or protein function [4, 5]. We, and others have employed proteomics-based methods to identify novel binding partners of proteins involved in specific cellular processes by using affinity purification coupled to mass spectrometry (AP-MS) (examples in references [6–9]). Each AP-MS experiment generates large amounts of data, which need to be thoroughly analysed in order to maximise the use of the entire dataset and to reach meaningful conclusions. Therefore data storage, its accessibility to the scientific community, and the ability to compare datasets has become a pressing issue. Here, we describe a *da*tabase for *p*rotein-*p*rotein int*er*actions (DAPPER) created to catalogue protein interaction networks of cell division regulators in *Drosophila melanogaster*. All the software and data contents are freely accessible through the web portal. Users can upload their own data and integrate with existing datasets through coordinating with DAPPER team. For sensitive/unpublished datasets, users are also welcome to download all of the DAPPER software as well as datasets on their local machine and integrate their datasets locally.

## Implementation

### Mass spectrometry-based analysis of protein-protein interactions

The detailed methodological description of the affinity purification of protein complexes from *Drosophila* cultured cells or *Drosophila* embryos for analysis by mass spectrometry was published previously [7, 9]. For immunoprecipitations using protein- or affinity-tag/epitope-specific antibodies, essentially the same protocol was followed as described in [7, 9], with few exceptions to accommodate different experimental requirements. Mass spectrometric analysis of purified protein samples was performed as published previously [10]. The raw data were successively analysed using the Mascot software and searched against the *Drosophila melanogaster* protein database obtained from FlyBase (www.flybase.org). Importantly, the last step of the analysis was the download of the data generated in each experiment using the “Export search results” function available on the Mascot Search Results web page (in the “Format As” section). The settings for the “Export search results” web page were as follows: Export format: “XML”; Significance threshold: “*p* < 0.05”; Ions score cut-off: “30”; Threshold type: “Identity”; Max. Number of hits: “AUTO”; Protein scoring: “MudPIT”; Include sub-set protein hits: “0”; Preferred taxonomy: “All entries”; and with checked boxes for the following parameters: Header, Decoy, Modification deltas, Search parameters, Format parameters, Score, Description, Mass (Da), Number of queries matched, Percent coverage, Experimental Mr (Da), Experimental charge, Calculated Mr (Da), Mass error (Da), Number of missed cleavages, Score, Expectation value, Sequence, Variable modifications, Query title. The XML-type files, which were generated during the download step, were then uploaded to DAPPER via the upload web page and further analysed using DAPPER-specific tools.

### Data deposition and system architecture

**Fig. 1:**
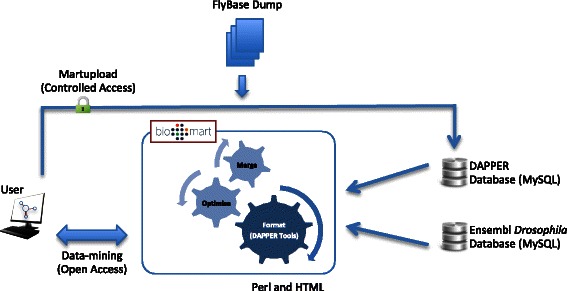
System-level architecture of DAPPER data-mining framework. End-user can either upload raw mascot XML files along with experimental annotations such as experimental conditions, or retrieve existing protein-protein/complex interactions. DAPPER contents are automatically annotated with FlyBase identifiers/links and further integrated with Ensembl *Drosophila melanogaster* database

While analyzing protein networks involved in *Drosophila* cell cycle regulation, we accumulated a large volume of proteomics data. Using these datasets, we created a database and a data-mining resource. This resource not only facilitates unified storage for lists of proteins identified during AP-MS experiments, but also allows cross comparison of individual datasets, and extraction of information difficult to mine otherwise. The DAPPER web interface (MartView) for querying protein-protein interaction datasets is available at: http://dapper.gen.cam.ac.uk/biomart/martview. The database is also made available through the BioMart Central Portal [11]. DAPPER is based on the BioMart data warehouse system version 0.7 [12, 13]; system-level view of DAPPER is shown in Fig. 1. A user can deposit data by uploading mascot XML files through Martupload utility or mine existing datasets using MartView utility. With regards to data uploads, each experiment is annotated with the bait and attributes such as affinity tags and drugs used during the purification and centrifugation settings. All experiments are automatically annotated with FlyBase Gene Identifiers and FlyBase Gene Names using the FlyBase data dumps [14]. The configuration of DAPPER enables automatic linking with a local copy of Ensembl *Drosophila melanogaster* database (version 75, BDGP5) [15]. DAPPER analytical tools offer useful views such as SORT, INTERSECTION, INTERSECTION PRIME and DISTINCT (Additional file 1: Figure S1). Briefly, SORT retrieves data sorted by the “Protein score” value, INTERSECTION retrieves proteins common to all selected experiments, INTERSECTION PRIME retrieves all entries that are not present in INTERSECTION, and DISTINCT retrieves entries that unique to a particular experiment. The DAPPER tools complement system’s mining abilities by enabling users to further prioritise hits in a meaningful way. Further, given the range of BioMart interoperable application programming interfaces (APIs) and software libraries including biomaRt (Bioconductor) [16], Galaxy [17], Taverna [18] and Cytoscape [19] users can seamlessly query DAPPER via BioMart Central Portal (www.biomart.org). Therefore, DAPPER contents are freely available to all the users of the aforementioned analytical platforms as well. DAPPER offers built-in integrative mining of Ensembl *Drosophila melanogaster* database annotations. A user query is split into DAPPER-specific attributes and Ensembl-specific attributes. Both databases are mined using MySQL queries independently, and results are integrated on-the-fly using *Drosophila* CG Identifiers*.* The data merging is performed in batches [13], and therefore results are returned as a continuous stream of aggregated records between the two data sources.

## Results and discussion

Presently, DAPPER contains data from 36 different cell cycle-related bait proteins (Additional file 2: Table S1) with a current coverage of 5,089 unique proteins (Additional file 3: Table S2). However, these numbers are increasing as more datasets are added to DAPPER on continual basis. The identified proteins, which were found interacting with the tested bait, are involved in many different biological processes predominantly focused on the proteins involved in cell cycle-related pathways. The following examples illustrate the data-mining capabilities and richness of DAPPER.

### Query 1

Here we give an example of how to mine DAPPER for the presence of a specific *Drosophila* protein either used as bait or identified as a prey. This search enables users to find a specific protein of interest in DAPPER. The query can be executed through DAPPER’s homepage by entering the CG identifier (e.g. *CG10722*) of the protein of interest and then clicking the “Search” button (Additional file 1: Figure S2A). CG numbers are unique identifiers for *Drosophila melanogaster* genes/proteins, which can be easily found by querying FlyBase (http://flybase.org). The query retrieves a list of pre-selected (default) attributes that are the properties of the protein encoded by *CG10722,* including the gene name(s) of its baits and FlyBase identifiers (Additional file 1: Figure S2B). Note that FlyBase Gene ID and Gene Name hyperlink to FlyBase and Ensembl web interfaces, respectively.

### Query 2

In this example, we demonstrate how a user can select all the proteins of interest identified by AP-MS that interact with a specific bait of interest. Following the “Browse DB” button on the home page (Additional file 1: Figure S2A), the user selects the database “Proteomics (Cambridge UK)” from the “Choose database” drop-down menu (Additional file 1: Figure S3A). Then in “Filters”, the user should select “Protein features”, enter the experiment identifier “12” in the section “Limit to experiment id/s”, and finally press “Results” on the top left (Additional file 1: Figure S3B). A table appears showing ten hits with the highest MASCOT protein score (the highest on the top) (Additional file 1: Figure S3C). Several pre-selected attributes (columns of the table) are listed in the table, including the number of peptides identified, the protein mass, and some FlyBase attributes. The user can increase the number of rows (and therefore the number of identified hits visible on the screen) in the “View” menu (Additional file 1: Figure S3D). The query can be modified at any time by changing the filters and attributes listed in the navigation panel on the left side of the MartView interface. Furthermore, it is also possible to integrate information from the Ensembl *Drosophila melanogaster* database through the “Dataset” option at the bottom left (Additional file 1: Figure S3E-F). The user can choose any combination of Ensembl attributes such as GO Term Name, GOA description, and Interpro Description, which are automatically mapped to results in DAPPER using the CG Gene ID as mapping entity (Additional file 1: Figure S3G).

### Query 3

Using DAPPER, users can also identify the proteins that are assigned with a specific Gene Ontology (GO) term in Ensembl from the entire mass spectrometric database. In the following example, the user identifies known components of the “kinetochore” (a proteinous cellular structure formed in the centromeric region of chromosomes) amongst all the proteins co-purified with a specific bait protein (e.g. Fzy, *CG4274*). The accession number for the Fzy protein, *CG4274*, is entered in the Filter Bait accession number (Additional file 1: Figure S4A). Next, the user chooses Ensembl as the additional dataset. New lists of additional Filters and Attributes will appear on the screen in the summary panel on the left hand side (Additional file 1: Figure S4B). In the additional Filters the user opens the GENE ONTOLOGY section, sets the GO Term Name to “kinetochore” (Additional file 1: Figure S4C), and hits Results. As shown in Additional file 1: Figure S4D, all the additional Attributes have been removed to simplify the search result and all the protein names are shown in “SORT_HTML” format. The user finds known kinetochore components that were identified in the purification of Fzy protein, including the bait protein itself. Amongst those hits, the user finds “BubR1” (*CG7838*), a protein involved in the spindle assembly checkpoint that was shown to directly bind Fzy and also to localise to kinetochores when the spindle assembly checkpoint is active [20, 21]. Interestingly, in the same query we also found “Spc105R” (*CG11451*), a kinetochore component involved in the recruitment of the SAC machinery via direct interaction. So far there has been no evidence of the direct interaction between Fzy and Spc105R. Thus, this result suggests a potential new kinetochore loading pathway for Fzy: Spc105R might recruit Fzy onto the kinetochore via the SAC. For additional example, please see Additional file (Additional file 1: Figure S4D).

DAPPER is content-rich and carefully curated with high-resolution experiments performed using affinity purifications followed by mass spectrometry. DAPPER is presented through widely accepted and interoperable web and programmatic access interface of BioMart software. In addition to the database contents, the web interface also enables analysis through several tools, which allow, for example sorting of identified proteins or eliminating common contaminants found by mass spectrometry. The database is also cross-linked with two other major *Drosophila* annotation resources, Ensembl and FlyBase. The contents are continually updated and will be further extended to incorporate protein-protein interactions in *Homo sapiens*. As the current DAPPER-Ensembl integration system facilitates this expansion effortlessly, we envisage that proteomic to genomic data integration for *Homo sapiens* would substantially enhance the value of DAPPER. Moreover, the analytical framework will be further extended by adding network analysis tools to interrogate DAPPER contents alongside other pathway databases such as REACTOME [22], KEGG [23, 24], NCI-PID [25] and Pathway commons [26].

DAPPER shares some features with other databases already available online. For example, The *Drosophila* Interaction Database, DroID (www.droidb.org) [27] also allows browsing and/or searching for interactions of a chosen protein/bait. Additionally, it includes the data on protein-miRNA and genetic interactions, and the database is directly linked to FlyBase (www.flybase.org) [14]. However, each database varies in its content, and contains different and often complementary data through *ad hoc* analytical tools. In contrast, DAPPER is extremely flexible and extendable. The open source software and database readily enables integration of new mass spectrometry datasets with existing ones; either in-house private or public datasets. Furthermore, DAPPER exploits BioMart’s user-friendly interface enabling powerful queries. This makes DAPPER unique compared to other experimentally validated protein-interaction databases of *Drosophila melanogaster*.

The data deposited in DAPPER originated from studies targeting cell cycle-relevant proteins and protein complexes and therefore the database is particularly useful for researchers interested in cell cycle regulation. As demonstrated in Query 3, DAPPER can facilitate the characterisation of unknown regulatory cascades of the proteins of interest. However, the contents of DAPPER are increasingly growing beyond cell cycle interactions, with various other bait such as chromatin-related proteins and associated interactions [1], phosphoprotein phosphatases and their regulators or substrates [28].

## Conclusions

DAPPER is a collection of a large number of proteomic datasets never previously released. Beyond an *ad-hoc* database, DAPPER is also an analytically unique method of mining and integrating previously published datasets alongside new experiments. In future, we intend to extend DAPPER to human protein-protein interactions and federate these with Ensembl *Homo sapiens* database.

## Availability and requirements

Project name: DAPPER

Project home page: http://dapper.gen.cam.ac.uk/

Operating systems: Windows, Unix/Linux, Mac OSX

Programming languages: Perl, Java script, HTML

Other requirements: none

License: GNU Lesser General Public License (LGPL) v3

Any restrictions to use by non-academics: none

## Additional files

Additional file 1: Figure S1. An overview of DAPPER retrieval tools. Venn diagram highlighting sections of records which will be retrieved by various tools, when applied to three hypothetical experiments. Each of the three tools can retrieve data in either tab-delimited or HTML format (see DAPPER Results web page for these options). **Figure S2.** Home page of DAPPER. The search field can be used to retrieve all experiments containing a particular CGID of *Drosophila melanogaster*. Browse button (MartView link) navigates to DAPPER BioMart interface for custom querying. By default, results are sorted (descending) by “Protein score”. Upload link is currently restricted to DAPPER curators and collaborators only. **Figure S3.** Stepwise guide to retrieve all proteins and their ontological annotations for a given experiment. The guide also shows how data from an external resource (Ensembl) can be retrieved and seamlessly integrated with DAPPER contents. **Figure S4.** Stepwise guide to retrieve kinetochore associated *Drosophila* genes. Gene ontology filter (GO Term Name) is set to “kinetochore” through linking with Ensembl database. Entries retrieved are further sorted by “Protein score” using SORT tool (SORT_HTML) on the Results page. **Figure S5.** Stepwise guide to compare two (or more) experiments. The results will contain only those hits which are common between the experiments selected for comparison (BubR1 and Polo kinase). Experiments having an entry in common are grouped together for better interpretation of results. (PDF 2334 kb) Additional file 2: Table S1. List of unique Baits in DAPPER. (XLSX 31 kb) Additional file 3: Table S2. List of unique CGIDs in DAPPER. Uniprot ID is shown where CGID was not available. (XLSX 92 kb)

## Abbreviations

AP-MS: Affinity-purification mass spectrometry
Da: Dalton
DAPPER: Database of protein-protein interactions
GO: Gene ontology
KEGG: Kyoto encyclopedia of genes and genomes
MS: Mass spectrometry
NCI-PID: National cancer institute – Pathway interaction database
