# Supplementary material for: DAPPER: a data-mining resource for protein-protein interactions
Source: BioData Min. 2015 Sep 24;8:30. doi: 10.1186/s13040-015-0063-3 (PMC4581157; doi:10.1186/s13040-015-0063-3)
Supplement: Additional file 1: Figure S1. — An overview of DAPPER retrieval tools. Venn diagram highlighting sections of records which will be retrieved by various tools, when applied to three hypothetical experiments. Each of the three tools can retrieve data in either tab-delimited or HTML format (see DAPPER Results web page for these options). Figure S2. Home page of DAPPER. The search field can be used to retrieve all experiments containing a particular CGID of Drosophila melanogaster. Browse button (MartView link) navigates to DAPPER BioMart interface for custom querying. By default, results are sorted (descending) by “Protein score”. Upload link is currently restricted to DAPPER curators and collaborators only. Figure S3. Stepwise guide to retrieve all proteins and their ontological annotations for a given experiment. The guide also shows how data from an external resource (Ensembl) can be retrieved and seamlessly integrated with DAPPER contents. Figure S4. Stepwise guide to retrieve kinetochore associated Drosophila genes. Gene ontology filter (GO Term Name) is set to “kinetochore” through linking with Ensembl database. Entries retrieved are further sorted by “Protein score” using SORT tool (SORT_HTML) on the Results page. Figure S5. Stepwise guide to compare two (or more) experiments. The results will contain only those hits which are common between the experiments selected for comparison (BubR1 and Polo kinase). Experiments having an entry in common are grouped together for better interpretation of results. (PDF 2334 kb) [file 13040_2015_63_MOESM1_ESM.pdf]

SF 1

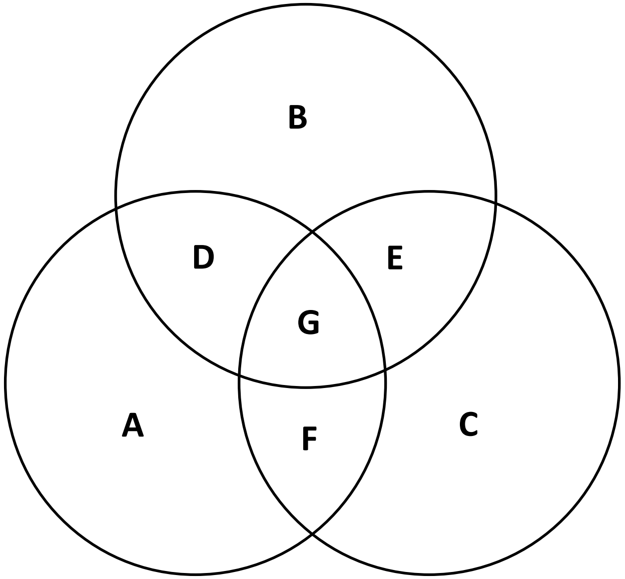

INTERSECTION = {*G*}
INTERSECTION PRIME = {*A, B, C, D, E, F*}
DISTINCT = {*A, B, C*}

SF 2A

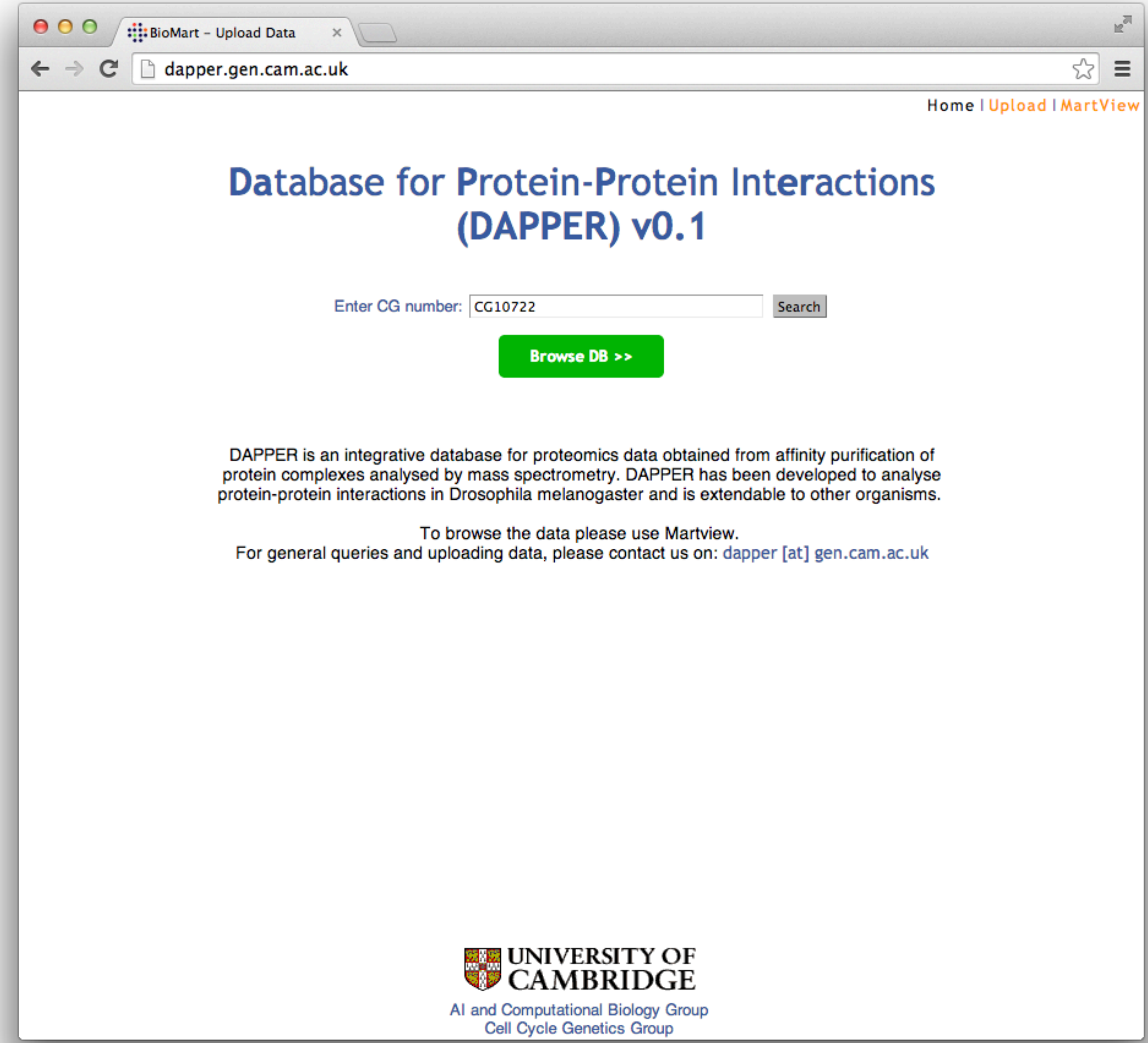
BioMart – Upload Data
dapper.gen.cam.ac.uk
Home | Upload | MartView
Database for Protein-Protein Interactions (DAPPER) v0.1
Enter CG number:
CG10722
Search
Browse DB >>
DAPPER is an integrative database for proteomics data obtained from affinity purification of protein complexes analysed by mass spectrometry. DAPPER has been developed to analyse protein-protein interactions in Drosophila melanogaster and is extendable to other organisms.
To browse the data please use Martview.
For general queries and uploading data, please contact us on: dapper [at] gen.cam.ac.uk
UNIVERSITY OF CAMBRIDGE
AI and Computational Biology Group
Cell Cycle Genetics Group

SF 2B

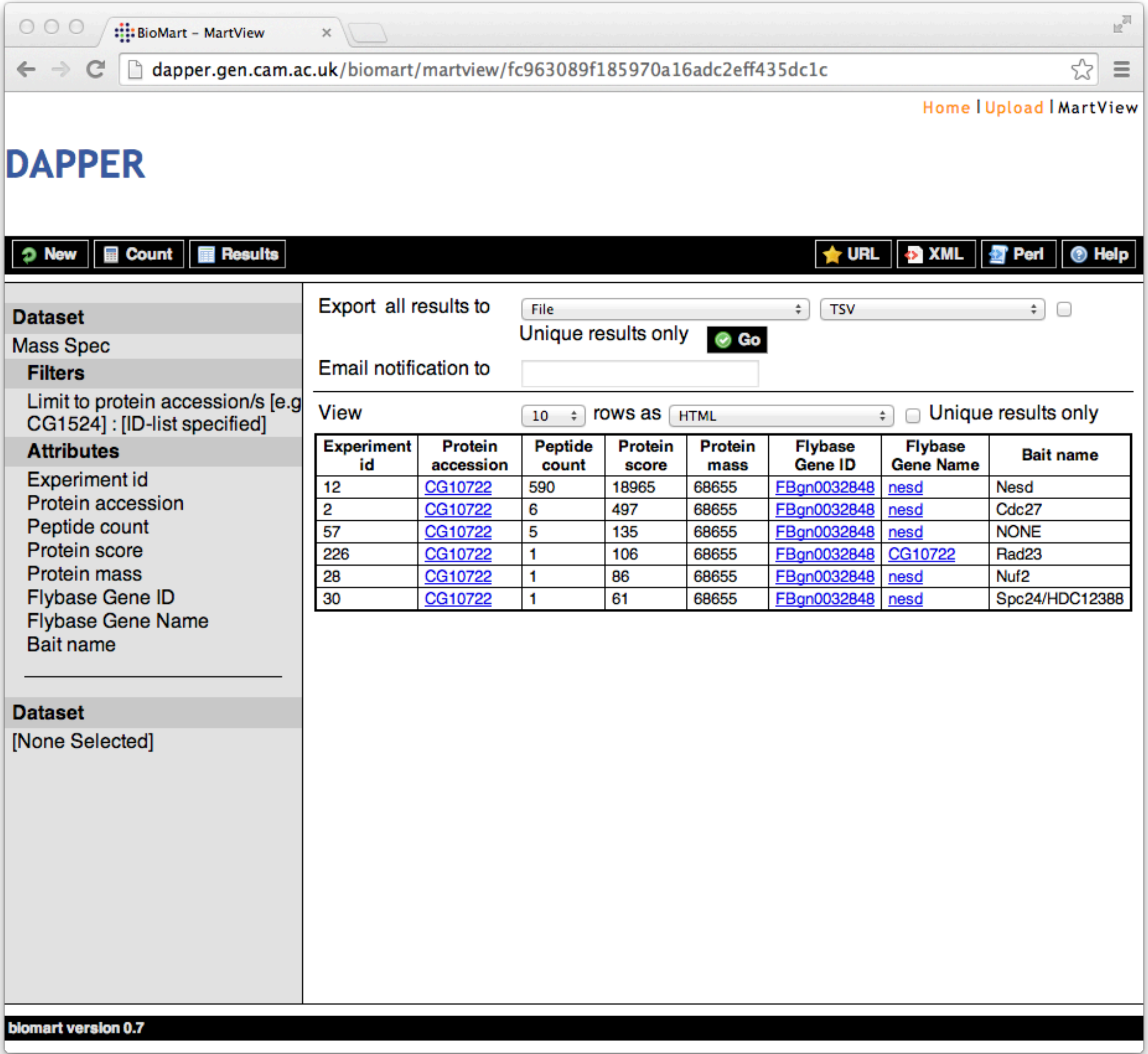

Experiment id	Protein accession	Peptide count	Protein score	Protein mass	Flybase Gene ID	Flybase Gene Name	Bait name
12	CG10722	590	18965	68655	FBgn0032848	nesd	Nesd
2	CG10722	6	497	68655	FBgn0032848	nesd	Cdc27
57	CG10722	5	135	68655	FBgn0032848	nesd	NONE
226	CG10722	1	106	68655	FBgn0032848	CG10722	Rad23
28	CG10722	1	86	68655	FBgn0032848	nesd	Nuf2
30	CG10722	1	61	68655	FBgn0032848	nesd	Spc24/HDC12388

SF 3A

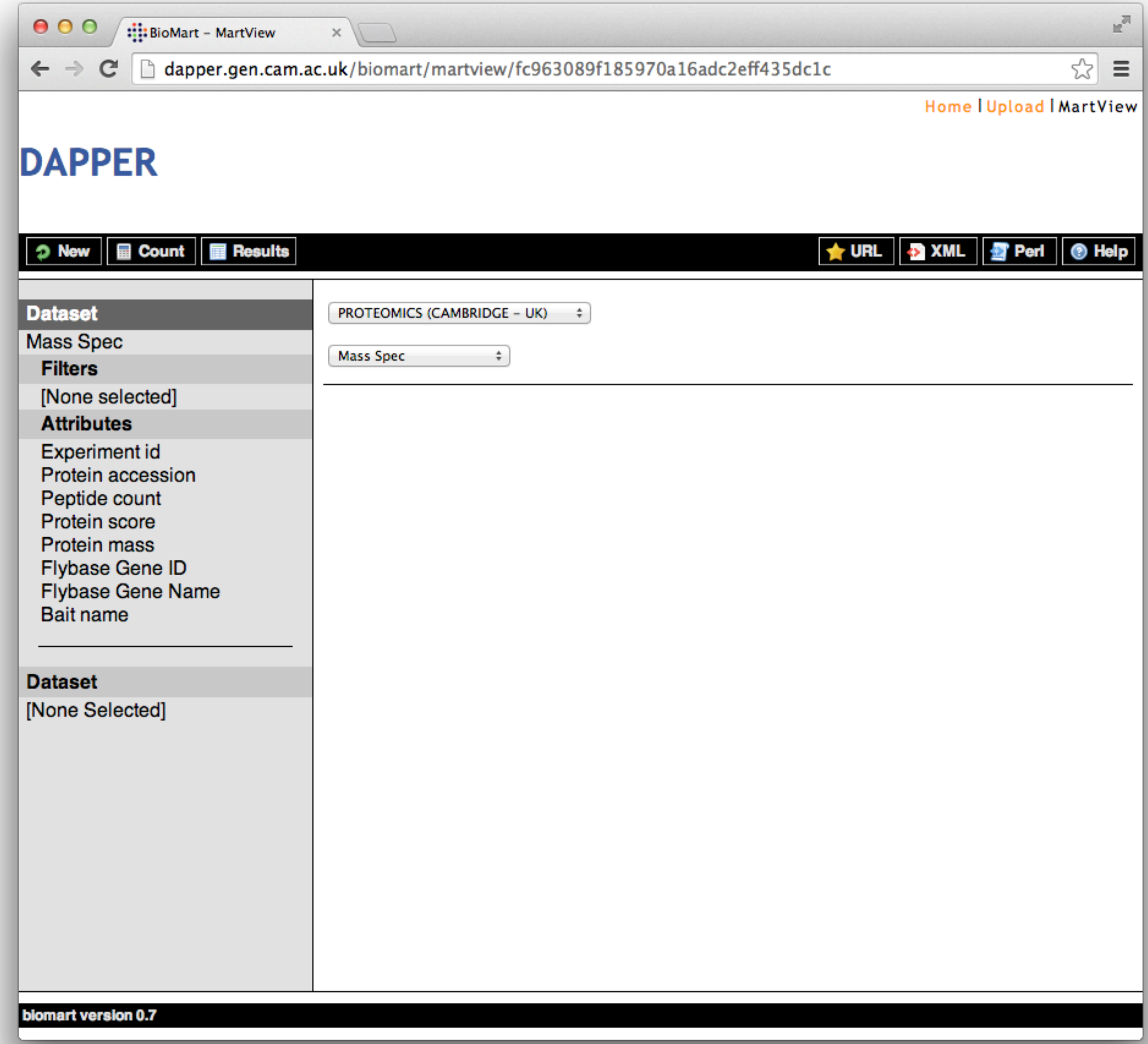
BioMart – MartView
dapper.gen.cam.ac.uk/biomart/martview/fc963089f185970a16adc2eff435dc1c
Home | Upload | MartView
DAPPER
New
Count
Results
URL
XML
Perl
Help
Dataset
Mass Spec
Filters
[None selected]
Attributes
Experiment id
Protein accession
Peptide count
Protein score
Protein mass
Flybase Gene ID
Flybase Gene Name
Bait name
Dataset
[None Selected]
PROTEOMICS (CAMBRIDGE – UK)
Mass Spec
biomart version 0.7

SF 3B

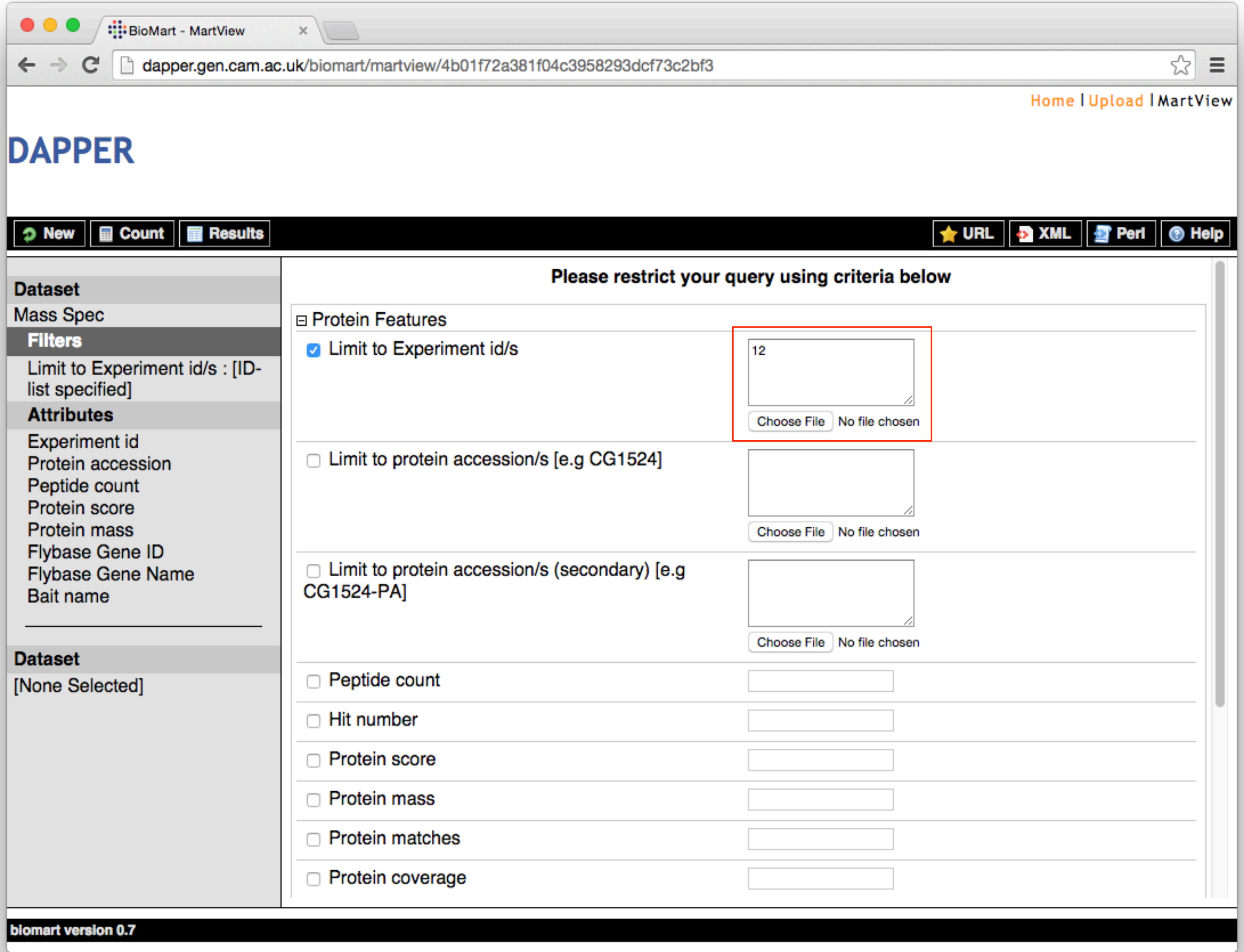
BioMart - MartView
dapper.gen.cam.ac.uk/biomart/martview/4b01f72a381f04c3958293dcf73c2bf3
Home | Upload | MartView
DAPPER
New
Count
Results
URL
XML
Perl
Help
Dataset
Mass Spec
Filters
Limit to Experiment id/s : [ID-list specified]
Attributes
Experiment id
Protein accession
Peptide count
Protein score
Protein mass
Flybase Gene ID
Flybase Gene Name
Bait name
Dataset
[None Selected]
Please restrict your query using criteria below
Protein Features
Limit to Experiment id/s
12
Choose File
No file chosen
Limit to protein accession/s [e.g CG1524]
Choose File
No file chosen
Limit to protein accession/s (secondary) [e.g CG1524-PA]
Choose File
No file chosen
Peptide count
Hit number
Protein score
Protein mass
Protein matches
Protein coverage
biomart version 0.7

SF 3C

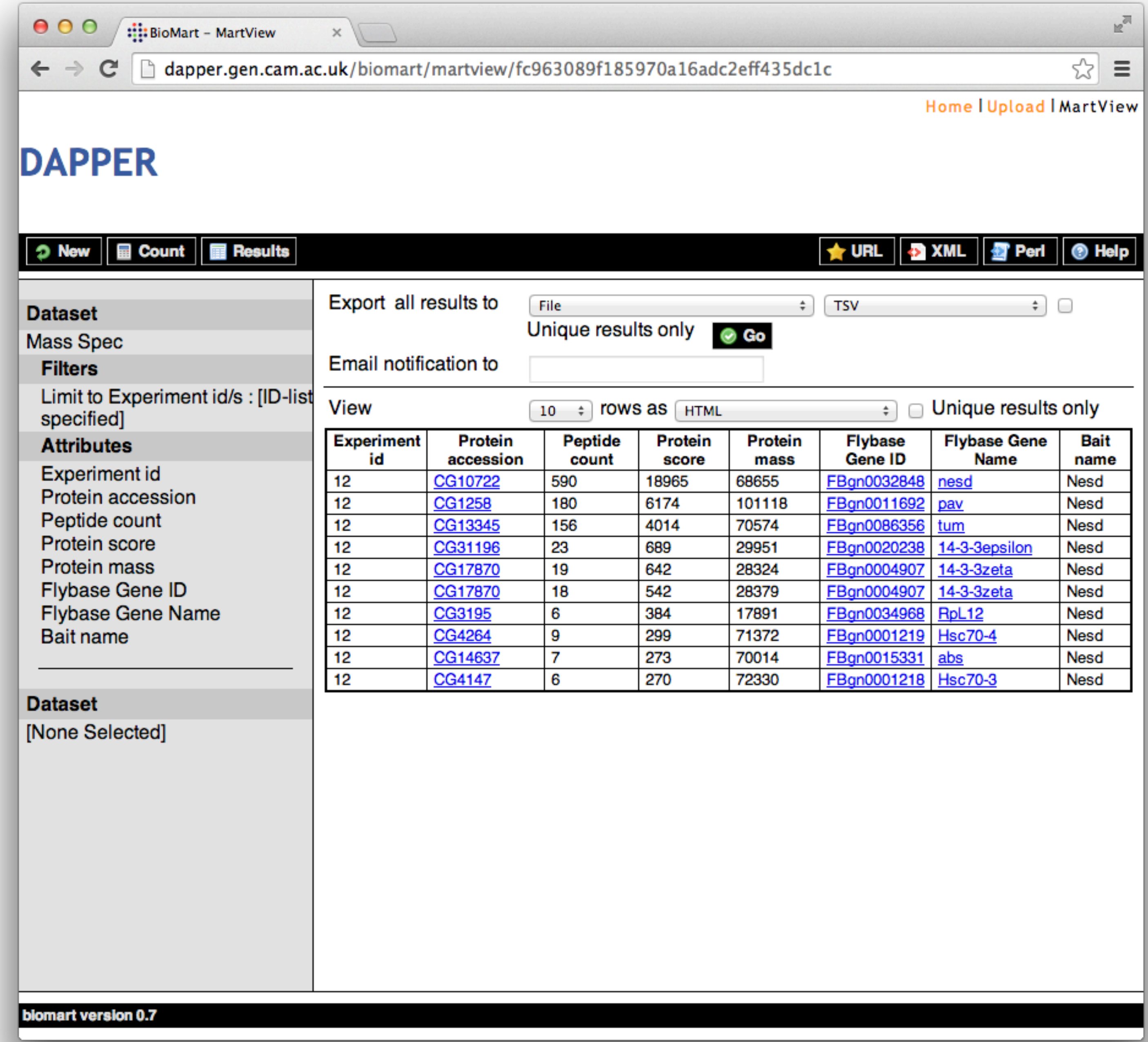

BioMart – MartView

dapper.gen.cam.ac.uk/biomart/martview/fc963089f185970a16adc2eff435dc1c

Home | Upload | MartView

DAPPER

New | Count | Results | URL | XML | Perl | Help

Dataset

Mass Spec

Filters

Limit to Experiment id/s : [ID-list specified]

Attributes

Experiment id
Protein accession
Peptide count
Protein score
Protein mass
Flybase Gene ID
Flybase Gene Name
Bait name

Dataset

[None Selected]

Export all results to File TSV Unique results only Go

Email notification to

View 10 rows as HTML Unique results only

Experiment id	Protein accession	Peptide count	Protein score	Protein mass	Flybase Gene ID	Flybase Gene Name	Bait name
12	CG10722	590	18965	68655	FBgn0032848	nesd	Nesd
12	CG1258	180	6174	101118	FBgn0011692	pav	Nesd
12	CG13345	156	4014	70574	FBgn0086356	tum	Nesd
12	CG31196	23	689	29951	FBgn0020238	14-3-3epsilon	Nesd
12	CG17870	19	642	28324	FBgn0004907	14-3-3zeta	Nesd
12	CG17870	18	542	28379	FBgn0004907	14-3-3zeta	Nesd
12	CG3195	6	384	17891	FBgn0034968	RpL12	Nesd
12	CG4264	9	299	71372	FBgn0001219	Hsc70-4	Nesd
12	CG14637	7	273	70014	FBgn0015331	abs	Nesd
12	CG4147	6	270	72330	FBgn0001218	Hsc70-3	Nesd

biomart version 0.7

SF 3D

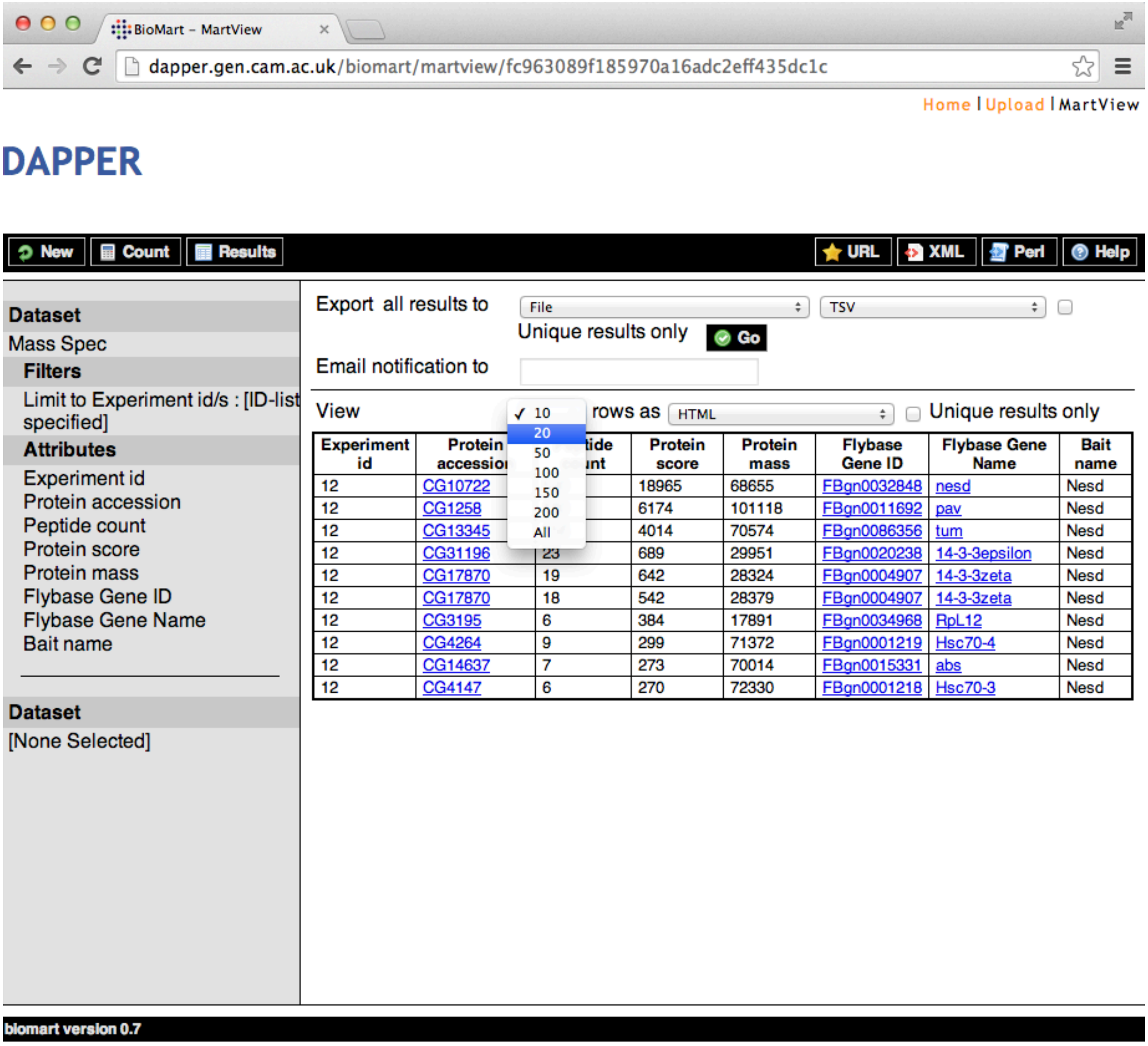
BioMart – MartView
dapper.gen.cam.ac.uk/biomart/martview/fc963089f185970a16adc2eff435dc1c
Home | Upload | MartView
DAPPER
New
Count
Results
URL
XML
Perl
Help
Dataset
Mass Spec
Filters
Limit to Experiment id/s : [ID-list specified]
Attributes
Experiment id
Protein accession
Peptide count
Protein score
Protein mass
Flybase Gene ID
Flybase Gene Name
Bait name
Dataset
[None Selected]
Export all results to
File
TSV
Unique results only
Go
Email notification to
View
10
20
50
100
150
200
All
rows as
HTML
Unique results only
Experiment id
Protein accession
Protein score
Protein mass
Flybase Gene ID
Flybase Gene Name
Bait name
12 CG10722 18965 68655 FBgn0032848 nesd Nesd
12 CG1258 6174 101118 FBgn0011692 pav Nesd
12 CG13345 4014 70574 FBgn0086356 tum Nesd
12 CG31196 23 689 29951 FBgn0020238 14-3-3epsilon Nesd
12 CG17870 19 642 28324 FBgn0004907 14-3-3zeta Nesd
12 CG17870 18 542 28379 FBgn0004907 14-3-3zeta Nesd
12 CG3195 6 384 17891 FBgn0034968 RpL12 Nesd
12 CG4264 9 299 71372 FBgn0001219 Hsc70-4 Nesd
12 CG14637 7 273 70014 FBgn0015331 abs Nesd
12 CG4147 6 270 72330 FBgn0001218 Hsc70-3 Nesd
biomart version 0.7

SF 3E

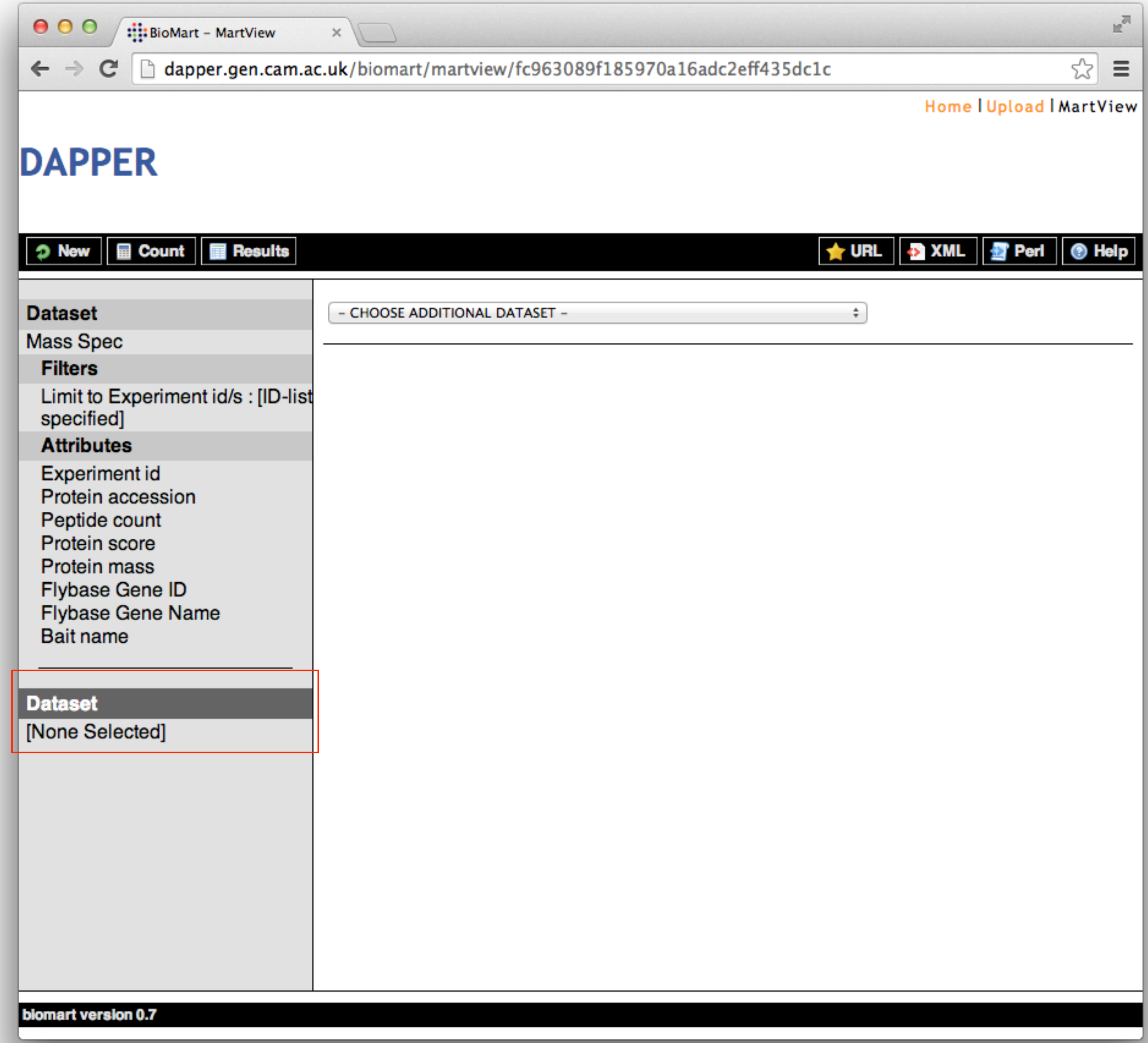
BioMart – MartView
dapper.gen.cam.ac.uk/biomart/martview/fc963089f185970a16adc2eff435dc1c
Home | Upload | MartView
DAPPER
New
Count
Results
URL
XML
Perl
Help
Dataset
Mass Spec
Filters
Limit to Experiment id/s : [ID-list specified]
Attributes
Experiment id
Protein accession
Peptide count
Protein score
Protein mass
Flybase Gene ID
Flybase Gene Name
Bait name
Dataset
[None Selected]
- CHOOSE ADDITIONAL DATASET -
biomart version 0.7

SF 3F

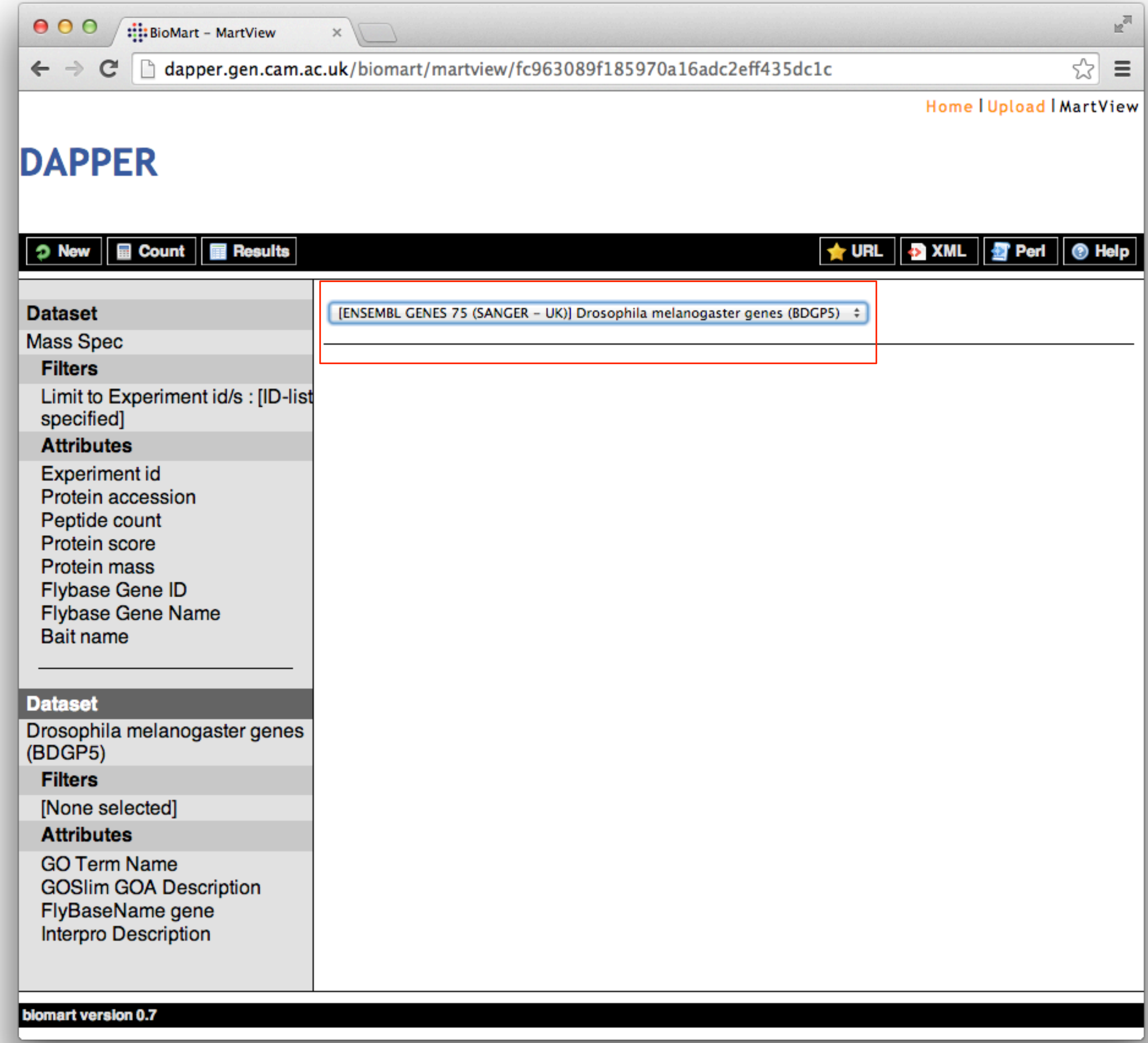
BioMart – MartView
dapper.gen.cam.ac.uk/biomart/martview/fc963089f185970a16adc2eff435dc1c
Home | Upload | MartView
DAPPER
New
Count
Results
URL
XML
Perl
Help
[ENSEMBL GENES 75 (SANGER – UK)] Drosophila melanogaster genes (BDGP5)
Dataset
Mass Spec
Filters
Limit to Experiment id/s : [ID-list specified]
Attributes
Experiment id
Protein accession
Peptide count
Protein score
Protein mass
Flybase Gene ID
Flybase Gene Name
Bait name
Dataset
Drosophila melanogaster genes (BDGP5)
Filters
[None selected]
Attributes
GO Term Name
GOSlim GOA Description
FlyBaseName gene
Interpro Description
biomart version 0.7

SF 3G

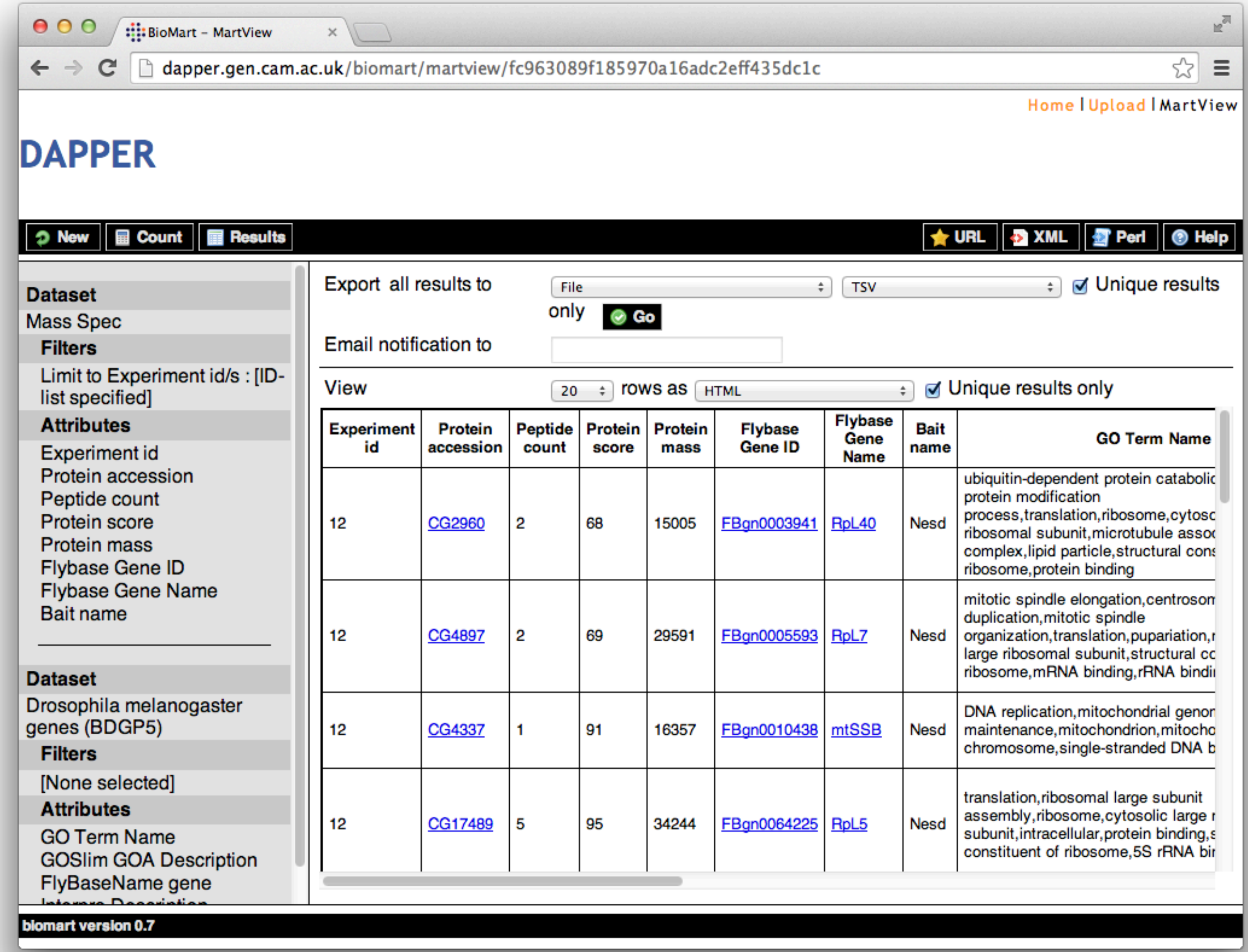
BioMart – MartView
dapper.gen.cam.ac.uk/biomart/martview/fc963089f185970a16adc2eff435dc1c
Home | Upload | MartView
DAPPER
New
Count
Results
URL
XML
Perl
Help
Dataset
Mass Spec
Filters
Limit to Experiment id/s : [ID-list specified]
Attributes
Experiment id
Protein accession
Peptide count
Protein score
Protein mass
Flybase Gene ID
Flybase Gene Name
Bait name
Dataset
Drosophila melanogaster genes (BDGP5)
Filters
[None selected]
Attributes
GO Term Name
GOSlim GOA Description
FlyBaseName gene
Export all results to
File
TSV
Unique results only
Go
Email notification to
View
20
rows as
HTML
Unique results only
Experiment id
Protein accession
Peptide count
Protein score
Protein mass
Flybase Gene ID
Flybase Gene Name
Bait name
GO Term Name
12 CG2960 2 68 15005 FBgn0003941 RpL40 Nesd
ubiquitin-dependent protein catabolic protein modification process,translation,ribosome,cytosc ribosomal subunit,microtubule assoc complex,lipid particle,structural cons ribosome,protein binding
12 CG4897 2 69 29591 FBgn0005593 RpL7 Nesd
mitotic spindle elongation,centrosom duplication,mitotic spindle organization,translation,pupariation,r large ribosomal subunit,structural cc ribosome,mRNA binding,rRNA bindi
12 CG4337 1 91 16357 FBgn0010438 mtSSB Nesd
DNA replication,mitochondrial genor maintenance,mitochondrion,mitocho chromosome,single-stranded DNA b
12 CG17489 5 95 34244 FBgn0064225 RpL5 Nesd
translation,ribosomal large subunit assembly,ribosome,cytosolic large r subunit,intracellular,protein binding,s constituent of ribosome,5S rRNA bi
biomart version 0.7

SF 4A

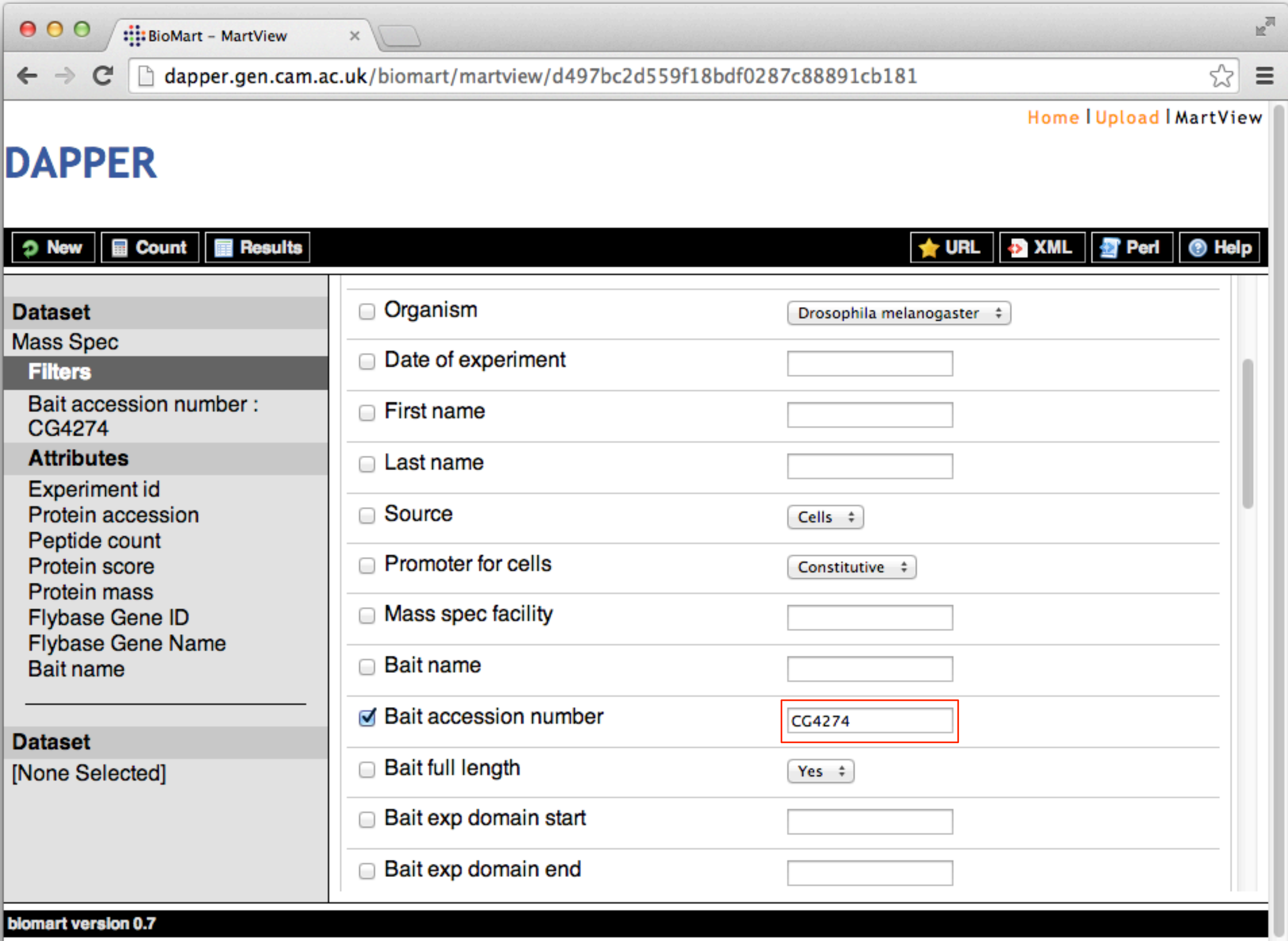
BioMart – MartView
dapper.gen.cam.ac.uk/biomart/martview/d497bc2d559f18bdf0287c88891cb181
Home | Upload | MartView
DAPPER
New
Count
Results
URL
XML
Perl
Help
Dataset
Mass Spec
Filters
Bait accession number : CG4274
Attributes
Experiment id
Protein accession
Peptide count
Protein score
Protein mass
Flybase Gene ID
Flybase Gene Name
Bait name
Dataset
[None Selected]
Organism
Drosophila melanogaster
Date of experiment
First name
Last name
Source
Cells
Promoter for cells
Constitutive
Mass spec facility
Bait name
Bait accession number
CG4274
Bait full length
Yes
Bait exp domain start
Bait exp domain end
biomart version 0.7

SF 4B

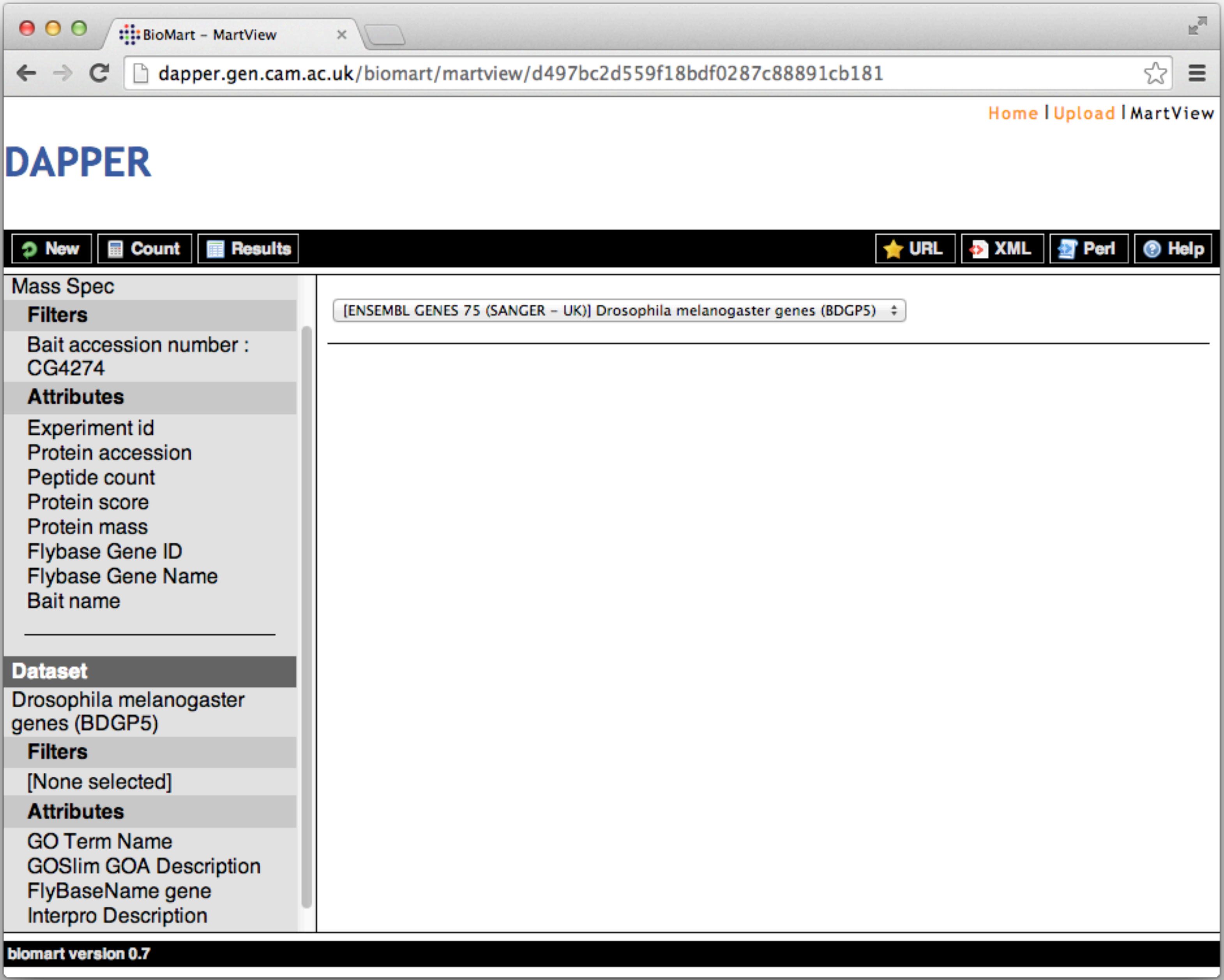
BioMart – MartView
dapper.gen.cam.ac.uk/biomart/martview/d497bc2d559f18bdf0287c88891cb181
Home | Upload | MartView
DAPPER
New
Count
Results
URL
XML
Perl
Help
Mass Spec
Filters
Bait accession number : CG4274
Attributes
Experiment id
Protein accession
Peptide count
Protein score
Protein mass
Flybase Gene ID
Flybase Gene Name
Bait name
Dataset
Drosophila melanogaster genes (BDGP5)
Filters
[None selected]
Attributes
GO Term Name
GOSlim GOA Description
FlyBaseName gene
Interpro Description
[ENSEMBL GENES 75 (SANGER – UK)] Drosophila melanogaster genes (BDGP5)
biomart version 0.7

SF 4C

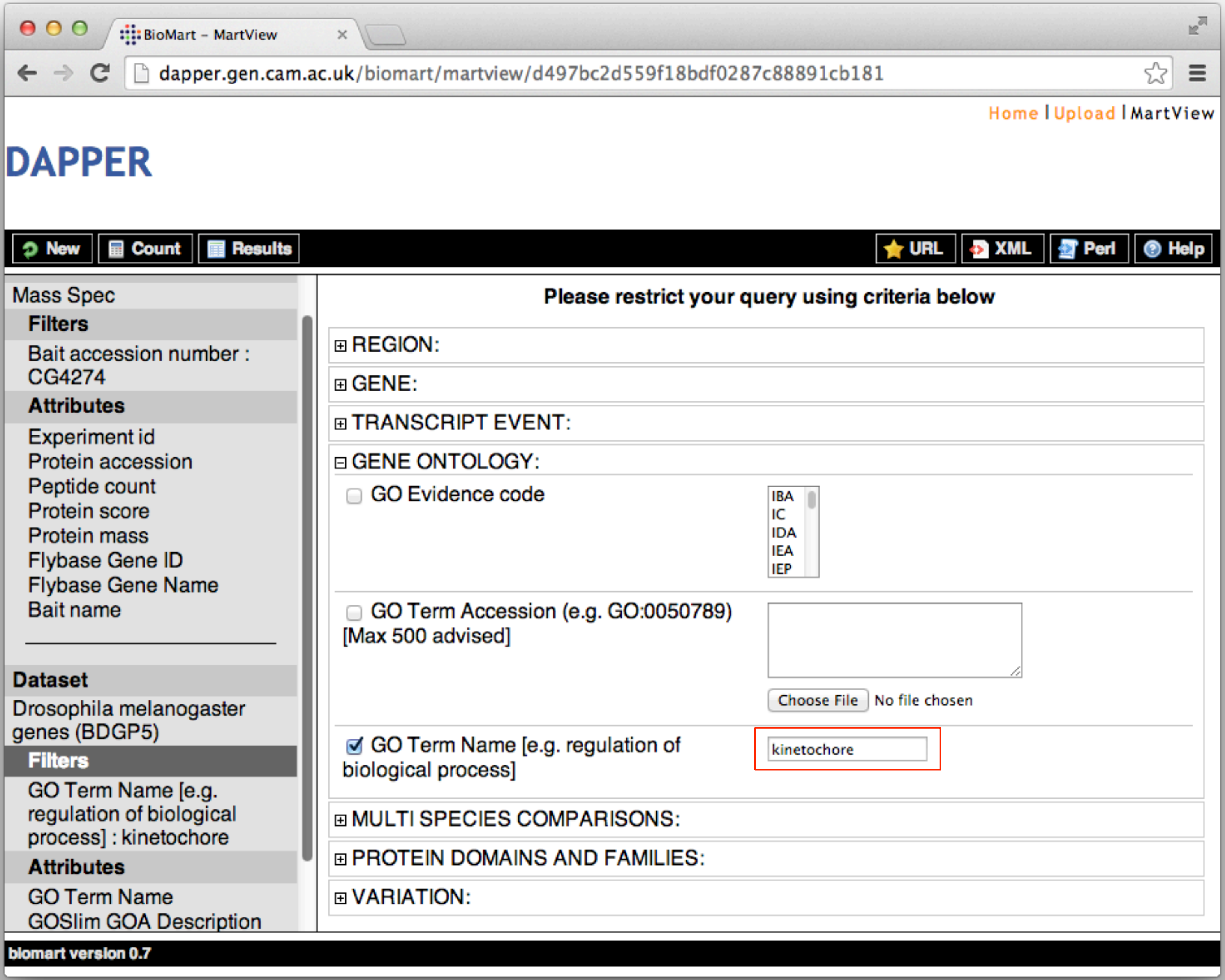
BioMart – MartView
dapper.gen.cam.ac.uk/biomart/martview/d497bc2d559f18bdf0287c88891cb181
Home | Upload | MartView
DAPPER
New
Count
Results
URL
XML
Perl
Help
Mass Spec
Filters
Bait accession number : CG4274
Attributes
Experiment id
Protein accession
Peptide count
Protein score
Protein mass
Flybase Gene ID
Flybase Gene Name
Bait name
Dataset
Drosophila melanogaster genes (BDGP5)
Filters
GO Term Name [e.g. regulation of biological process] : kinetochore
Attributes
GO Term Name
GOSlim GOA Description
Please restrict your query using criteria below
REGION:
GENE:
TRANSCRIPT EVENT:
GENE ONTOLOGY:
GO Evidence code
IBA
IC
IDA
IEA
IEP
GO Term Accession (e.g. GO:0050789) [Max 500 advised]
Choose File
No file chosen
GO Term Name [e.g. regulation of biological process]
kinetochore
MULTI SPECIES COMPARISONS:
PROTEIN DOMAINS AND FAMILIES:
VARIATION:
biomart version 0.7

SF 4D

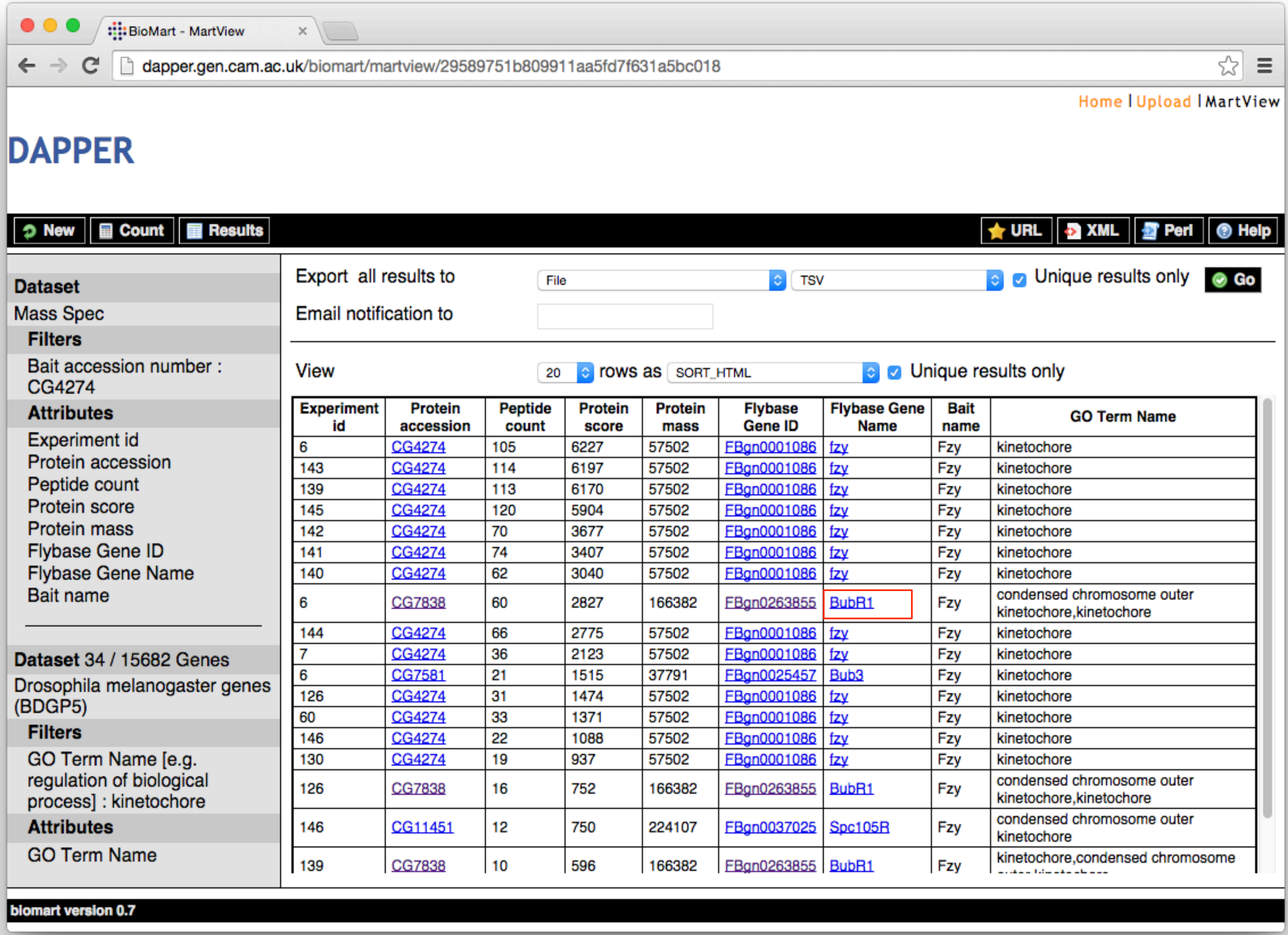
BioMart - MartView

dapper.gen.cam.ac.uk/biomart/martview/29589751b809911aa5fd7f631a5bc018

Home | Upload | MartView

DAPPER

New | Count | Results | URL | XML | Perl | Help

Dataset
Mass Spec
Filters
Bait accession number : CG4274
Attributes
Experiment id
Protein accession
Peptide count
Protein score
Protein mass
Flybase Gene ID
Flybase Gene Name
Bait name

Dataset 34 / 15682 Genes
Drosophila melanogaster genes (BDGP5)
Filters
GO Term Name [e.g. regulation of biological process] : kinetochore
Attributes
GO Term Name

Export all results to File TSV Unique results only Go

Email notification to

View 20 rows as SORT_HTML Unique results only

Experiment id	Protein accession	Peptide count	Protein score	Protein mass	Flybase Gene ID	Flybase Gene Name	Bait name	GO Term Name
6	CG4274	105	6227	57502	FBgn0001086	fzy	Fzy	kinetochore
143	CG4274	114	6197	57502	FBgn0001086	fzy	Fzy	kinetochore
139	CG4274	113	6170	57502	FBgn0001086	fzy	Fzy	kinetochore
145	CG4274	120	5904	57502	FBgn0001086	fzy	Fzy	kinetochore
142	CG4274	70	3677	57502	FBgn0001086	fzy	Fzy	kinetochore
141	CG4274	74	3407	57502	FBgn0001086	fzy	Fzy	kinetochore
140	CG4274	62	3040	57502	FBgn0001086	fzy	Fzy	kinetochore
6	CG7838	60	2827	166382	FBgn0263855	BubR1	Fzy	condensed chromosome outer kinetochore,kinetochore
144	CG4274	66	2775	57502	FBgn0001086	fzy	Fzy	kinetochore
7	CG4274	36	2123	57502	FBgn0001086	fzy	Fzy	kinetochore
6	CG7581	21	1515	37791	FBgn0025457	Bub3	Fzy	kinetochore
126	CG4274	31	1474	57502	FBgn0001086	fzy	Fzy	kinetochore
60	CG4274	33	1371	57502	FBgn0001086	fzy	Fzy	kinetochore
146	CG4274	22	1088	57502	FBgn0001086	fzy	Fzy	kinetochore
130	CG4274	19	937	57502	FBgn0001086	fzy	Fzy	kinetochore
126	CG7838	16	752	166382	FBgn0263855	BubR1	Fzy	condensed chromosome outer kinetochore,kinetochore
146	CG11451	12	750	224107	FBgn0037025	Spc105R	Fzy	condensed chromosome outer kinetochore
139	CG7838	10	596	166382	FBgn0263855	BubR1	Fzy	kinetochore,condensed chromosome [illegible]

biomart version 0.7

SF 5A

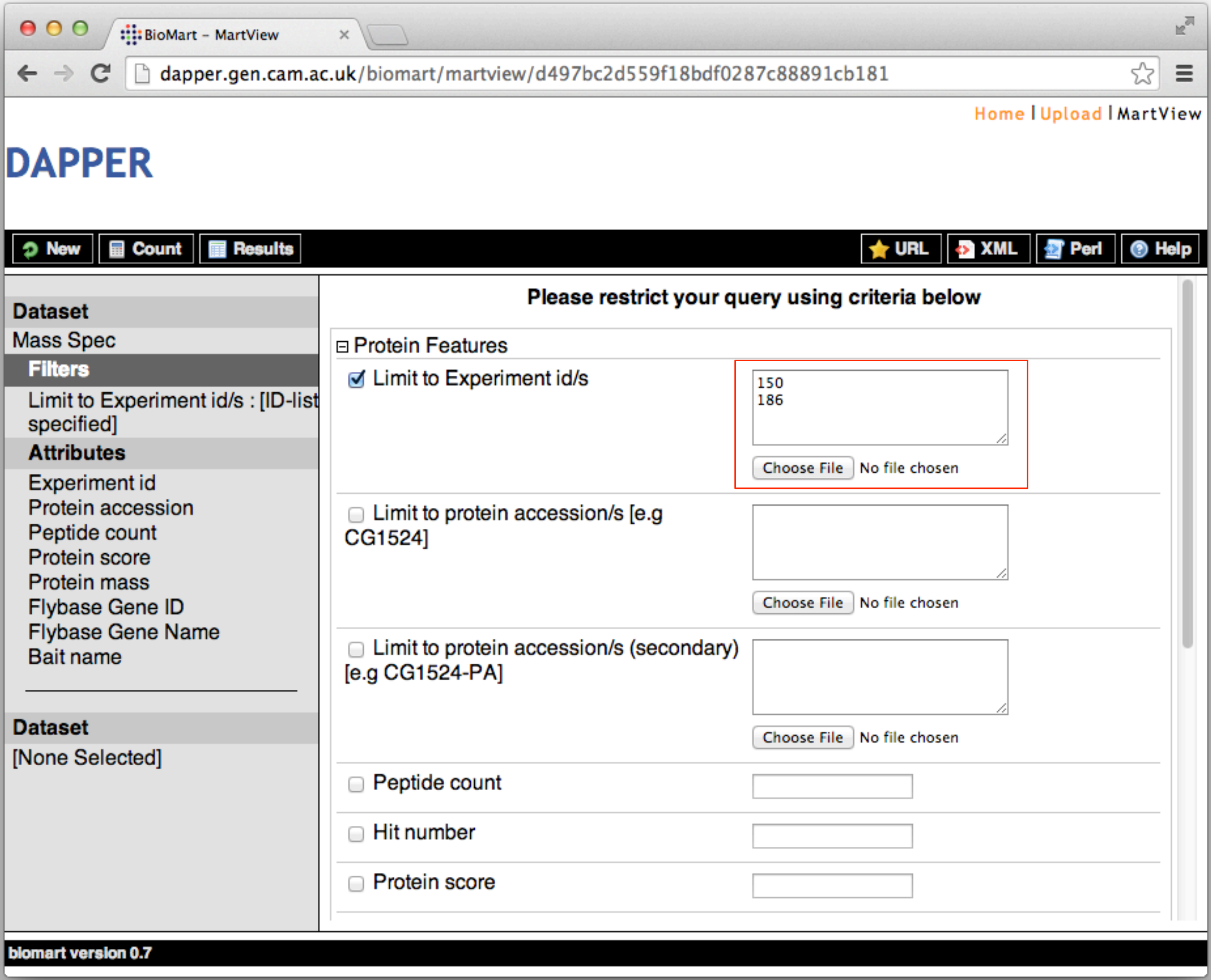

BioMart – MartView
dapper.gen.cam.ac.uk/biomart/martview/d497bc2d559f18bdf0287c88891cb181
Home | Upload | MartView
DAPPER
New
Count
Results
URL
XML
Perl
Help
Dataset
Mass Spec
Filters
Limit to Experiment id/s : [ID-list specified]
Attributes
Experiment id
Protein accession
Peptide count
Protein score
Protein mass
Flybase Gene ID
Flybase Gene Name
Bait name
Dataset
[None Selected]
Please restrict your query using criteria below
Protein Features
Limit to Experiment id/s
150
186
Choose File
No file chosen
Limit to protein accession/s [e.g CG1524]
Choose File
No file chosen
Limit to protein accession/s (secondary) [e.g CG1524-PA]
Choose File
No file chosen
Peptide count
Hit number
Protein score
biomart version 0.7

SF 5B

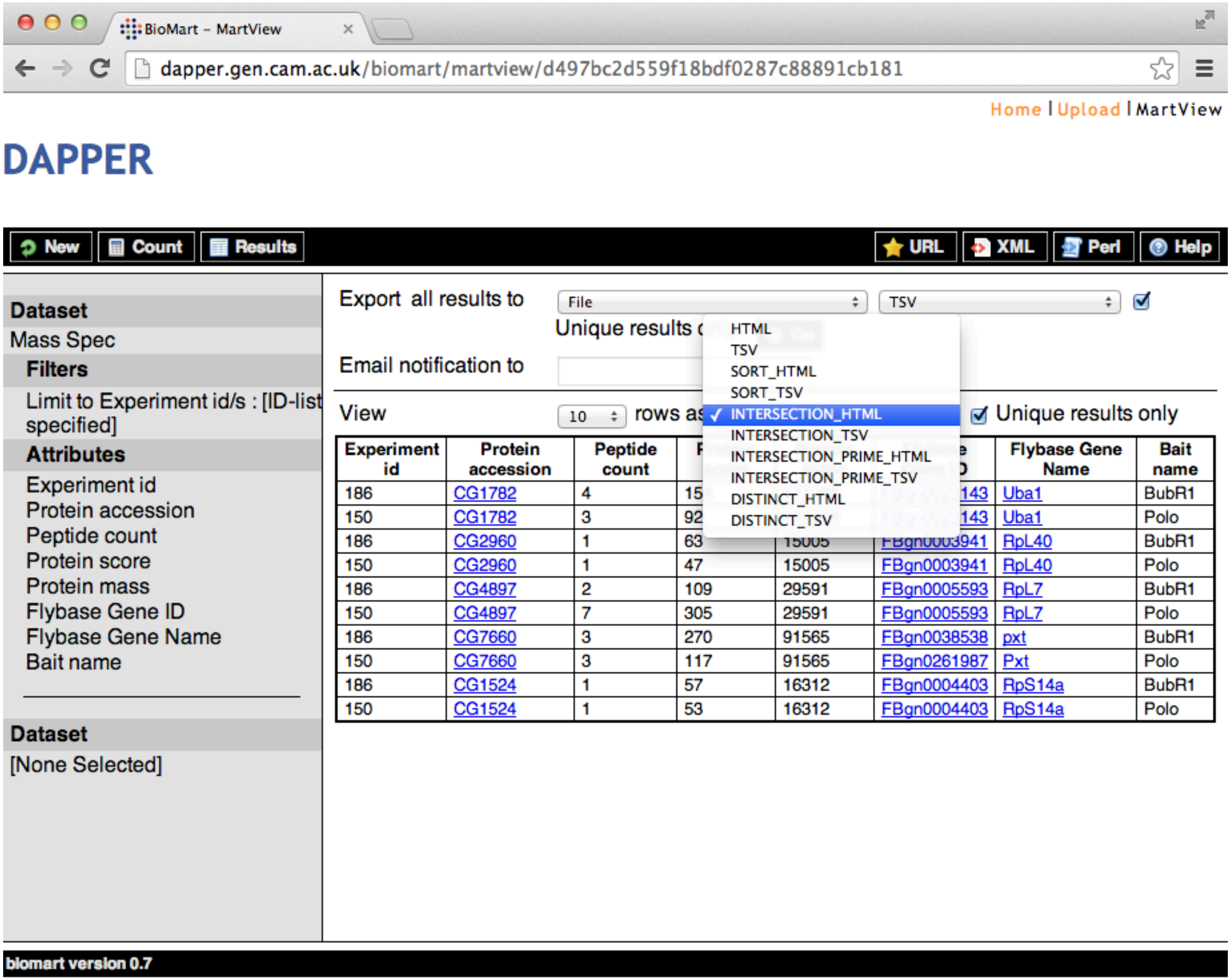
BioMart – MartView
dapper.gen.cam.ac.uk/biomart/martview/d497bc2d559f18bdf0287c88891cb181
Home | Upload | MartView
DAPPER
New
Count
Results
URL
XML
Perl
Help
Dataset
Mass Spec
Filters
Limit to Experiment id/s : [ID-list specified]
Attributes
Experiment id
Protein accession
Peptide count
Protein score
Protein mass
Flybase Gene ID
Flybase Gene Name
Bait name
Dataset
[None Selected]
Export all results to
File
TSV
Unique results
Email notification to
View
10
rows as
Unique results only
HTML
TSV
SORT_HTML
SORT_TSV
INTERSECTION_HTML
INTERSECTION_TSV
INTERSECTION_PRIME_HTML
INTERSECTION_PRIME_TSV
DISTINCT_HTML
DISTINCT_TSV
Experiment id
Protein accession
Peptide count
Flybase Gene Name
Bait name
186 CG1782 4 15 143 Uba1 BubR1
150 CG1782 3 92 143 Uba1 Polo
186 CG2960 1 63 FBgn0003941 RpL40 BubR1
150 CG2960 1 47 15005 FBgn0003941 RpL40 Polo
186 CG4897 2 109 29591 FBgn0005593 RpL7 BubR1
150 CG4897 7 305 29591 FBgn0005593 RpL7 Polo
186 CG7660 3 270 91565 FBgn0038538 pxt BubR1
150 CG7660 3 117 91565 FBgn0261987 Pxt Polo
186 CG1524 1 57 16312 FBgn0004403 RpS14a BubR1
150 CG1524 1 53 16312 FBgn0004403 RpS14a Polo
biomart version 0.7

SF 5C

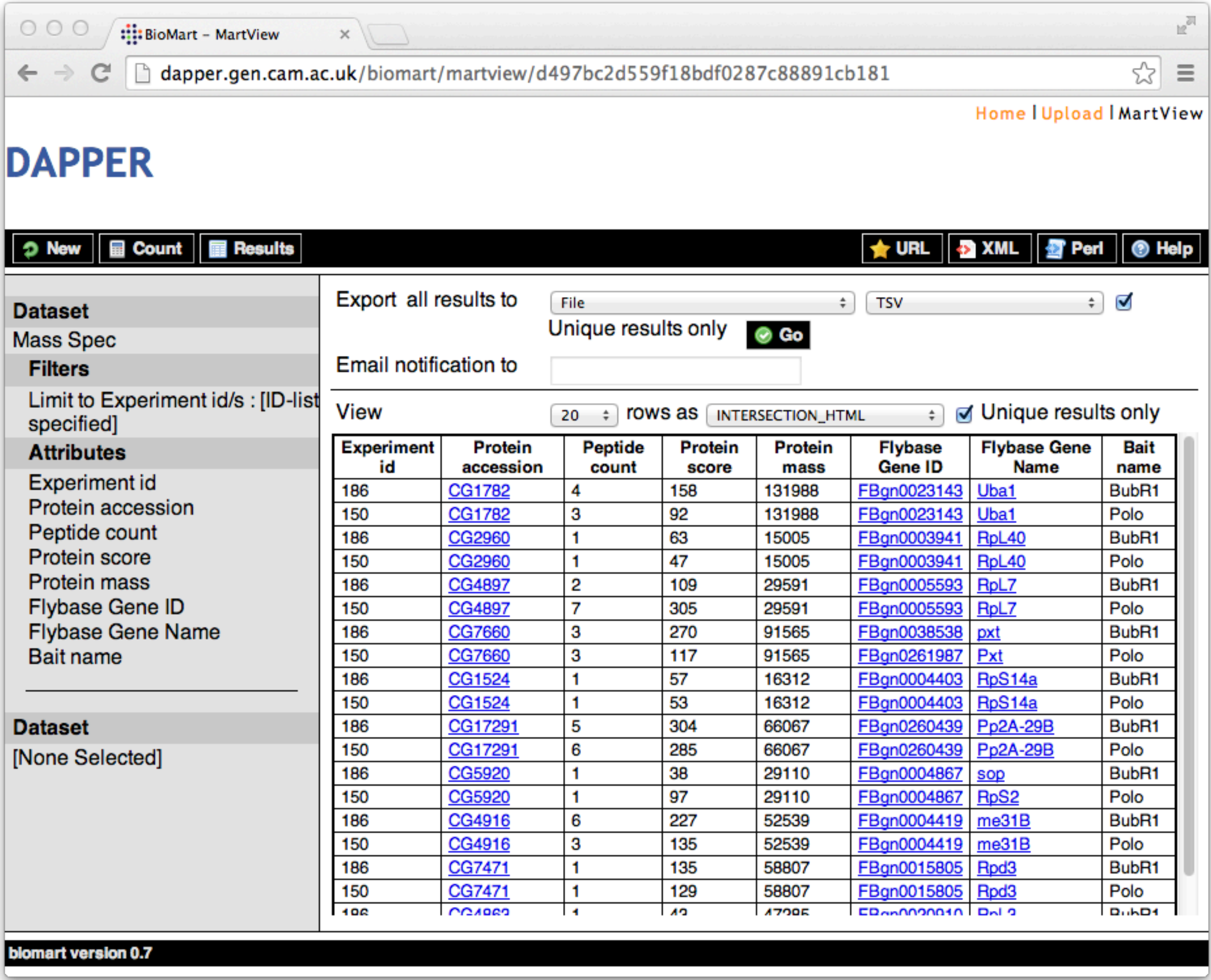

Experiment id	Protein accession	Peptide count	Protein score	Protein mass	Flybase Gene ID	Flybase Gene Name	Bait name
186	CG1782	4	158	131988	FBgn0023143	Uba1	BubR1
150	CG1782	3	92	131988	FBgn0023143	Uba1	Polo
186	CG2960	1	63	15005	FBgn0003941	RpL40	BubR1
150	CG2960	1	47	15005	FBgn0003941	RpL40	Polo
186	CG4897	2	109	29591	FBgn0005593	RpL7	BubR1
150	CG4897	7	305	29591	FBgn0005593	RpL7	Polo
186	CG7660	3	270	91565	FBgn0038538	pxt	BubR1
150	CG7660	3	117	91565	FBgn0261987	Pxt	Polo
186	CG1524	1	57	16312	FBgn0004403	RpS14a	BubR1
150	CG1524	1	53	16312	FBgn0004403	RpS14a	Polo
186	CG17291	5	304	66067	FBgn0260439	Pp2A-29B	BubR1
150	CG17291	6	285	66067	FBgn0260439	Pp2A-29B	Polo
186	CG5920	1	38	29110	FBgn0004867	sop	BubR1
150	CG5920	1	97	29110	FBgn0004867	RpS2	Polo
186	CG4916	6	227	52539	FBgn0004419	me31B	BubR1
150	CG4916	3	135	52539	FBgn0004419	me31B	Polo
186	CG7471	1	135	58807	FBgn0015805	Rpd3	BubR1
150	CG7471	1	129	58807	FBgn0015805	Rpd3	Polo
[illegible]	[illegible]	[illegible]	[illegible]	[illegible]	[illegible]	[illegible]	[illegible]
